# Firearm Homicides and Suicides in Major Metropolitan Areas — United States, 2006–2007 and 2009–2010

**Published:** 2013-08-02

**Authors:** Scott R. Kegler, James A. Mercy

**Affiliations:** Div of Analysis, Research, and Practice Integration; Div of Violence Prevention, National Center for Injury Prevention and Control, CDC

Firearm homicides and suicides are a continuing public health concern in the United States. During 2009–2010, a total of 22,571 firearm homicides and 38,126 firearm suicides occurred among U.S. residents (1). This includes 3,397 firearm homicides and 1,548 firearm suicides among persons aged 10–19 years; the firearm homicide rate for this age group was slightly above the all-ages rate. This report updates an earlier report* that provided statistics on firearm homicides and suicides in major metropolitan areas for 2006–2007, with special emphasis on persons aged 10–19 years in recognition of the importance of early prevention efforts. Firearm homicide and suicide rates were calculated for the 50 most populous U.S. metropolitan statistical areas (MSAs)† for 2009–2010 using mortality data from the National Vital Statistics System (NVSS) and population data from the U.S. Census Bureau. Comparison statistics were recalculated for 2006–2007 to reflect revisions to MSA delineations and population estimates subsequent to the earlier report. Although the firearm homicide rate for large MSAs collectively remained above the national rate during 2009–2010, more than 75% of these MSAs showed a decreased rate from 2006–2007, largely accounting for a national decrease. The firearm homicide rate for persons aged 10–19 years exceeded the all-ages rate in many of these MSAs during 2009–2010, similar to the earlier reporting period. Conversely, although the firearm suicide rate for large MSAs collectively remained below the national rate during 2009–2010, nearly 75% of these MSAs showed an increased rate from 2006–2007, paralleling the national trend. Firearm suicide rates among persons aged 10–19 years were low compared with all-ages rates during both periods. These patterns can inform the development and monitoring of strategies directed at reducing firearm-related violence.

NVSS mortality data for 2006–2007 and 2009–2010 (the most recent available) were used to identify deaths attributed to firearm homicides (*International Classification of Diseases, 10th Revision* [ICD-10] underlying cause codes X93–X95 and U01.4 [provisional]) and firearm suicides (codes X72–X74) among U.S. residents. Firearm homicide and suicide counts were tabulated for county groupings forming the 50 largest MSAs (by population rank mid-year 2010).§ Tabulated counts were integrated with U.S. Census Bureau population estimates for the counties forming these MSAs to calculate annual firearm homicide and suicide rates for persons of all ages (excluding those aged <10 years for suicides because intent for self-harm typically is not attributed to young children). Rates were calculated similarly for persons aged 10–19 years. All-ages rates were age-adjusted to the year 2000 U.S. standard. MSA-level data involving firearm homicide or suicide counts <20 are not reported separately because of concerns about statistical stability and data privacy. However, such data were included in the calculations for all MSAs combined.

All-ages firearm homicide rates during 2009–2010 varied widely by MSA, ranging from 1.1 to 19.0 per 100,000 residents per year (Table). The rate for all MSAs combined was 4.3, compared with a national rate of 3.7. This represents a decrease from 2006–2007, when the combined MSA rate was 5.2 and the national rate was 4.2. Firearm homicide rates decreased for 78% of MSAs (39 of 50) across reporting periods, accounting for most of the national decrease. The firearm homicide rate among persons aged 10–19 years for the MSAs collectively was 5.1 during 2009–2010. This also reflects a decrease from 2006–2007, when the combined MSA rate for persons aged 10–19 years was 6.6. Rates for this age group exceeded all-ages rates in 72% of MSAs during 2009–2010 (23 of 32 MSAs with reportable youth firearm homicide statistics), comparable to the percentage observed for the earlier period. Males accounted for approximately 85% of firearm homicide victims (all ages) during both reporting periods, for all MSAs combined as well as nationally.

All-ages firearm suicide rates during 2009–2010 also varied widely by MSA, ranging from 1.6 to 11.4 (Table). The combined MSA rate was 5.4, compared with a national rate of 7.0. This represents an increase from 2006–2007, when the combined MSA rate was 5.1 and the national rate was 6.5. Across reporting periods, firearm suicide rates increased for 74% of MSAs (37 of 50), mirroring the national trend. Firearm suicide rates among persons aged 10–19 years were low compared with all-ages rates; the combined MSA rate for this age group was 1.2 during both reporting periods. Males represented approximately 87% of firearm suicides (all ages) in both reporting periods for all MSAs combined and nationally.

## Editorial Note

During 2009–2010, homicide was the 15th leading cause of death (all ages) in the United States and the second leading cause among persons aged 10–19 years; a firearm injury was the underlying cause in 68% of all homicides and in 83% of homicides among youths (1). The findings in this report show that despite declining firearm homicide rates in most large metropolitan areas, rates collectively remained higher in these areas compared with the United States overall. Residents of the 50 largest MSAs represented 54% of the U.S. population during 2009–2010 (unchanged from 2006–2007) but accounted for 64% of firearm homicide victims nationally (somewhat below the percentage for 2006–2007). These MSAs accounted for 70% of the national firearm homicide total (2,368 of 3,397) among persons aged 10–19 years.

Concurrently, suicide was the 10th leading cause of death (all ages) nationally and the third leading cause for persons aged 10–19 years; a firearm injury was the underlying cause in 51% of all suicides and in 40% of suicides among youths (1). Firearm suicide rates increased in most large metropolitan areas across reporting periods; however, rates collectively remained lower in these areas compared with the United States overall. Although residents of the large MSAs comprised more than half of the U.S. population, they accounted for just 42% of firearm suicides nationally (identical to the percentage for 2006–2007). For persons aged 10–19 years, these MSAs accounted for 37% of firearm suicides nationwide.

What is already known on this topic?

Firearm homicide rates for large metropolitan statistical areas (MSAs) have been found to be higher than for the United States overall, with rates also higher among persons aged 10–19 years than among persons of all ages. In contrast, firearm suicide rates have been found to be lower in these large urban areas than for the nation overall.

What is added by this report?

Although geographic and age-specific differences in firearm homicide rates have persisted, rates declined from 2006–2007 to 2009–2010 for most large MSAs, as well as nationally. The national decline in the firearm homicide rate can be attributed primarily to declines in these large metropolitan areas. Geographic differences in firearm suicide rates also have persisted; however, firearm suicide rates increased from 2006–2007 to 2009–2010 for most large MSAs and nationally.

What are the implications for public health practice?

Prevention and intervention research should focus on identifying effective strategies for sustaining declines in firearm homicide rates and stemming recent increases in firearm suicide rates. Although further study is needed, initiatives for reducing firearm-related violence can draw upon a growing evidence base for effectively addressing behavioral and environmental factors associated with both firearm and nonfirearm violence.

The findings in this report are subject to at least four limitations. First, statistics for central cities within MSAs are not presented because of lack of age-specific population estimates suitable for supporting rate comparisons across the periods considered. Second, statistics on nonfatal injuries associated with firearm assault or self-harm are not presented because population-based nonfatal injury data are not available for MSAs. Third, although the statistics for victims aged 10–19 years convey the serious impact of firearm-related violence on youths, other age groups not separately considered in this report had higher firearm homicide rates (e.g., persons aged 20–39 years, for whom rates have been declining recently) or higher firearm suicide rates (most or all other age groups, for whom rates variously have been level or increasing). Finally, the fraction of NVSS records with the underlying cause of death coded as “other ill-defined and unspecified causes of mortality” (ICD-10 code R99) was higher than usual for several states (New Jersey, Ohio, and West Virginia) and the District of Columbia for 2009. The influence of nonspecific cause codes on firearm fatality statistics is not known; however, the annual fraction of such records remained low (approximately 5% or less) for each of these states.

The observed declines in firearm homicide rates and increases in firearm suicide rates are consistent with longer-term trends in homicide and suicide nationally (1). Homicide rates generally have been declining in the United States during the past two decades (1). Factors identified by previous research as influencing this decline include shifting demographics, changes in markets for illegal drugs (e.g., type, demand, and participants), law enforcement responses to gun violence and drug-related crime, increased incarceration rates, community policing and related efforts, and improving economic conditions throughout much of the 1990s (2). Increasing suicide rates have been prominent in the middle-aged population during the past decade as the percentage of suicides accounted for by this group has steadily increased (1,3). Suicide rates within this age group previously have been associated with business cycles (4); national unemployment rates notably doubled from 2006–2007 to 2009–2010 (5).

A factor likely affecting firearm homicide and suicide is access to firearms by persons at risk for harming themselves or others. Potential strategies for reducing firearm-related violence among such persons include initiatives promoting safe storage of guns (6), waiting periods to reduce the consequences of impulsive suicidal behavior (7), designing firearms to make them safer (8), and efforts such as background checks to prevent high-risk persons from possessing firearms (e.g., persons convicted of violent crimes, persons subject to protective orders because of threats of domestic violence, and persons with documented mental illness posing a risk to themselves or others) (9). Further research is needed to assess the effectiveness of such strategies.

Effective approaches for preventing violence include early education through school-based programs addressing social, emotional, and behavioral competencies; parent and family-based programs promoting positive relationships, communication, support, and proper supervision; and efforts to improve school, neighborhood, and community environments in ways that reduce the likelihood of violence (10).¶ Promoting the capacity of communities to implement such approaches might prove essential to achieving population-level impacts.

## Figures and Tables

**TABLE:** Numbers and annual rates (per 100,000 population) of firearm homicides and suicides for the 50 most populous metropolitan statistical areas (MSAs), by selected age groups — United States, 2006–2007 and 2009–2010*

		Firearm homicides				Firearm suicides			
			
		All ages		Aged 10–19 yrs		Aged ≥10 yrs		Aged 10–19 yrs	
					
MSA (ordered alphabetically)	Years	No.†	Rate§	No.	Rate	No.†	Rate§	No.	Rate
**U.S. total**	2006–2007	25,406	4.2	4,166	4.9	34,232	6.5	1,446	1.7
	2009–2010	22,560	3.7	3,397	4.0	38,122	7.0	1,548	1.8
**MSA total (50 MSAs)**	2006–2007	17,149	5.2	3,060	6.6	14,253	5.1	570	1.2
	2009–2010	14,428	4.3	2,368	5.1	15,960	5.4	578	1.2
Atlanta – Sandy Springs – Roswell, GA	2006–2007	661	6.4	84	5.8	566	7.0	21	1.4
	2009–2010	515	4.8	64	4.2	672	7.8	26	1.7
Austin – Round Rock, TX	2006–2007	50	1.4	¶	¶	171	6.6	¶	¶
	2009–2010	66	1.7	¶	¶	187	6.9	¶	¶
Baltimore – Columbia – Towson, MD	2006–2007	543	10.3	96	12.7	235	5.0	¶	¶
	2009–2010	409	7.7	54	7.4	223	4.6	¶	¶
Birmingham – Hoover, AL	2006–2007	242	11.0	33	10.9	181	9.4	¶	¶
	2009–2010	186	8.4	¶	¶	216	10.7	¶	¶
Boston – Cambridge – Newton, MA-NH	2006–2007	167	1.9	40	3.3	141	1.7	¶	¶
	2009–2010	166	1.8	32	2.7	165	2.0	¶	¶
Buffalo – Cheektowaga – Niagara Falls, NY	2006–2007	111	5.2	26	8.1	77	3.8	¶	¶
	2009–2010	103	4.7	¶	¶	73	3.5	¶	¶
Charlotte – Concord – Gastonia, NC-SC	2006–2007	215	5.3	31	5.4	261	7.5	¶	¶
	2009–2010	190	4.4	27	4.4	305	8.0	¶	¶
Chicago – Naperville – Elgin, IL-IN-WI	2006–2007	1,152	6.0	253	9.3	491	3.1	24	0.9
	2009–2010	1,139	6.0	213	7.9	527	3.2	¶	¶
Cincinnati, OH-KY-IN	2006–2007	177	4.3	35	5.8	233	6.5	¶	¶
	2009–2010	140	3.4	¶	¶	246	6.6	¶	¶
Cleveland – Elyria, OH	2006–2007	215	5.6	27	4.5	198	5.3	¶	¶
	2009–2010	146	3.9	21	3.7	168	4.5	¶	¶
Columbus, OH	2006–2007	165	4.3	26	5.1	221	7.1	¶	¶
	2009–2010	174	4.4	29	5.5	219	6.7	¶	¶
Dallas – Fort Worth – Arlington, TX	2006–2007	540	4.3	70	3.9	628	6.5	32	1.8
	2009–2010	469	3.6	57	3.0	762	7.4	25	1.3
Denver – Aurora – Lakewood, CO	2006–2007	122	2.5	26	4.0	353	8.6	¶	¶
	2009–2010	117	2.3	¶	¶	375	8.6	¶	¶
Detroit – Warren – Dearborn, MI	2006–2007	792	9.5	117	9.0	436	5.6	¶	¶
	2009–2010	686	8.6	119	9.5	461	6.1	31	2.5
Hartford – West Hartford – East Hartford, CT	2006–2007	62	2.7	¶	¶	46	2.1	¶	¶
	2009–2010	75	3.3	¶	¶	78	3.5	¶	¶
Houston – The Woodlands – Sugar Land, TX	2006–2007	761	6.7	115	6.9	590	6.7	32	1.9
	2009–2010	701	5.8	83	4.7	730	7.7	39	2.2
Indianapolis – Carmel – Anderson, IN	2006–2007	218	6.0	29	5.6	217	7.0	¶	¶
	2009–2010	188	5.1	25	4.7	248	7.7	¶	¶
Jacksonville, FL	2006–2007	243	9.3	37	10.1	183	8.1	¶	¶
	2009–2010	198	7.4	26	7.1	255	10.8	¶	¶
Kansas City, MO-KS	2006–2007	226	6.0	40	7.3	257	7.7	¶	¶
	2009–2010	260	6.8	62	11.3	284	8.2	¶	¶
Las Vegas – Henderson – Paradise, NV	2006–2007	221	6.0	46	9.2	340	11.1	¶	¶
	2009–2010	164	4.2	24	4.5	376	11.4	¶	¶
Los Angeles – Long Beach – Anaheim, CA	2006–2007	1,612	6.0	410	10.7	687	3.2	¶	¶
	2009–2010	1,141	4.2	251	6.7	755	3.5	22	0.6
Louisville/Jefferson County, KY-IN	2006–2007	119	5.2	¶	¶	194	9.2	¶	¶
	2009–2010	105	4.4	¶	¶	202	9.1	¶	¶
Memphis, TN-MS-AR	2006–2007	298	11.4	47	11.7	176	8.2	¶	¶
	2009–2010	249	9.4	43	10.7	182	8.4	¶	¶
Miami – Fort Lauderdale – West Palm Beach, FL	2006–2007	657	6.3	112	7.8	547	5.4	¶	¶
	2009–2010	594	5.6	81	5.8	580	5.5	¶	¶
Milwaukee – Waukesha – West Allis, WI	2006–2007	182	5.9	44	9.9	125	4.8	¶	¶
	2009–2010	139	4.5	¶	¶	156	5.6	¶	¶
Minneapolis – St. Paul – Bloomington, MN-WI	2006–2007	119	1.8	25	2.7	261	4.7	¶	¶
	2009–2010	90	1.3	¶	¶	295	5.1	¶	¶
Nashville-Davidson – Murfreesboro – Franklin, TN	2006–2007	169	5.2	¶	¶	266	9.8	¶	¶
	2009–2010	158	4.6	27	6.0	251	8.7	¶	¶
New Orleans – Metairie, LA	2006–2007	491	23.2	89	30.0	167	8.7	¶	¶
	2009–2010	449	19.0	80	25.6	157	7.5	¶	¶
New York – Newark – Jersey City, NY-NJ-PA	2006–2007	1,233	3.2	208	4.0	533	1.6	¶	¶
	2009–2010	1,101	2.8	203	3.9	574	1.6	¶	¶
Oklahoma City, OK	2006–2007	104	4.2	20	6.1	160	7.8	¶	¶
	2009–2010	90	3.5	20	5.9	207	9.5	¶	¶
Orlando – Kissimmee – Sanford, FL	2006–2007	242	5.7	28	4.8	210	5.9	¶	¶
	2009–2010	151	3.4	¶	¶	269	7.1	¶	¶
Philadelphia – Camden – Wilmington, PA-NJ-DE-MD	2006–2007	899	7.7	166	9.7	483	4.6	¶	¶
	2009–2010	729	6.2	124	7.5	478	4.4	¶	¶
Phoenix – Mesa – Scottsdale, AZ	2006–2007	555	6.9	96	8.5	616	9.2	33	2.9
	2009–2010	331	4.0	39	3.2	688	9.8	28	2.3
Pittsburgh, PA	2006–2007	187	4.4	35	5.7	296	6.7	¶	¶
	2009–2010	192	4.5	31	5.3	298	6.8	¶	¶
Portland – Vancouver – Hillsboro, OR-WA	2006–2007	62	1.4	¶	¶	264	7.2	¶	¶
	2009–2010	66	1.4	¶	¶	302	7.8	¶	¶
Providence – Warwick, RI-MA	2006–2007	47	1.5	¶	¶	76	2.6	¶	¶
	2009–2010	56	1.8	¶	¶	90	3.1	¶	¶
Raleigh, NC	2006–2007	50	2.5	¶	¶	91	5.4	¶	¶
	2009–2010	52	2.3	¶	¶	100	5.4	¶	¶
Richmond, VA	2006–2007	175	7.4	35	10.5	162	7.9	¶	¶
	2009–2010	134	5.7	¶	¶	151	7.1	¶	¶
Riverside – San Bernardino – Ontario, CA	2006–2007	396	4.8	80	5.7	356	5.6	¶	¶
	2009–2010	283	3.3	56	3.9	366	5.3	¶	¶
Sacramento – Roseville – Arden-Arcade, CA	2006–2007	149	3.5	30	4.8	204	5.7	¶	¶
	2009–2010	132	3.0	23	3.7	231	6.0	¶	¶
St. Louis, MO-IL	2006–2007	393	7.3	83	10.5	334	6.8	¶	¶
	2009–2010	436	8.1	82	10.6	354	7.0	¶	¶
Salt Lake City, UT	2006–2007	44	2.0	¶	¶	137	8.6	¶	¶
	2009–2010	32	1.5	¶	¶	194	11.2	¶	¶
San Antonio – New Braunfels, TX	2006–2007	185	4.5	27	4.4	240	7.3	¶	¶
	2009–2010	155	3.6	27	4.2	267	7.4	¶	¶
San Diego – Carlsbad, CA	2006–2007	149	2.4	30	3.5	251	5.0	¶	¶
	2009–2010	75	1.1	¶	¶	282	5.2	¶	¶
San Francisco – Oakland – Hayward, CA	2006–2007	576	6.9	106	10.4	242	3.2	¶	¶
	2009–2010	439	5.2	101	9.9	325	4.2	¶	¶
San Jose – Sunnyvale – Santa Clara, CA	2006–2007	45	1.2	¶	¶	79	2.6	¶	¶
	2009–2010	48	1.3	¶	¶	106	3.3	¶	¶
Seattle – Tacoma – Bellevue, WA	2006–2007	158	2.3	24	2.8	346	6.0	¶	¶
	2009–2010	105	1.5	¶	¶	406	6.7	¶	¶
Tampa – St. Petersburg – Clearwater, FL	2006–2007	179	3.5	21	3.1	395	7.8	¶	¶
	2009–2010	161	3.1	26	3.8	490	9.1	¶	¶
Virginia Beach – Norfolk – Newport News, VA-NC	2006–2007	198	5.6	32	6.6	179	6.2	¶	¶
	2009–2010	203	5.7	29	6.2	216	7.4	¶	¶
Washington – Arlington – Alexandria, DC-VA-MD-WV	2006–2007	593	5.4	93	6.4	351	3.9	¶	¶
	2009–2010	440	3.8	76	5.1	418	4.3	¶	¶

* Numbers and rates reflect decedent’s place of residence, not place of occurrence. This table includes only the 50 most populous MSAs among the 381 U.S. MSAs currently delineated, and therefore cannot be used to establish comprehensive national rankings.

† These national and MSA-specific numbers correspond to age-adjusted rates and exclude a small fraction of records with undocumented decedent age (28 firearm homicides and seven firearm suicides).

§ Rates are age-adjusted to the year 2000 U.S. standard population.

¶ Entry suppressed because of statistical instability or data confidentiality concerns (both associated with small numbers).

## Footnotes

* Available at http://www.cdc.gov/mmwr/pdf/wk/mm6018.pdf.

† An MSA is defined by the U.S. Office of Management and Budget (OMB) as “a core area containing a substantial population nucleus, together with adjacent communities.” This report is based on the revised geographic delineations for MSAs issued by OMB in February 2013.

§ The same MSAs were the 50 most populous during both reporting periods; rankings by total population changed slightly.

¶ Additional information available at http://www.cdc.gov/violenceprevention/stryve/index.html and http://www.thecommunityguide.org/violence/schoolbasedprograms.html.
